# Cilostazol Alleviates NLRP3 Inflammasome–Induced Allodynia/Hyperalgesia in Murine Cerebral Cortex Following Transient Ischemia: Focus on TRPA1/Glutamate and Akt/Dopamine/BDNF/Nrf2 Trajectories

**DOI:** 10.1007/s12035-022-03024-w

**Published:** 2022-09-20

**Authors:** Omnia S. Zaki, Noha N. Nassar, Dalaal M. Abdallah, Marwa M. Safar, Reham A. Mohammed

**Affiliations:** 1 Department of Pharmacology and Toxicology, Faculty of Pharmacy, Modern University for Technology and Information, Cairo, Egypt; 2 Department of Pharmacology and Toxicology, Faculty of Pharmacy, Cairo University, Kasr El-Aini Street, Cairo, 11562 Egypt; 3 Department of Pharmacology and Biochemistry, Faculty of Pharmacy, The British University in Egypt, Cairo, Egypt

**Keywords:** Anterior cingulate cortex, GFAP, Hyperalgesia, Mechanical/cold allodynia, TRPA1

## Abstract

Global cerebral ischemia/reperfusion (I/R) provokes inflammation that augments neuropathic pain. Cilostazol (CLZ) has pleiotropic effects including neuroprotection in several ravaging central disorders; nonetheless, its potential role in transient central ischemic-induced allodynia and hyperalgesia has not been asserted before. Rats were allocated into 4 groups; sham, sham + CLZ, and 45 min-bilateral carotid occlusion followed by a 48 h-reperfusion period either with or without CLZ (50 mg/kg; p.o) post-treatment. CLZ prolonged latency of hindlimb withdrawal following von Frey filaments, 4 °C cold, and noxious mechanical stimulations. Histopathological alterations and the immunoexpression of glial fibrillary acidic protein induced by I/R were reduced by CLZ in the anterior cingulate cortex (ACC) area, while, CLZ enhanced intact neuronal count. Meanwhile, CLZ modulated cerebral cortical glutamate, dopamine neurotransmission, and transient receptor potential ankyrin 1 (TRPA1). CLZ anti-inflammatory potential was mediated by the downregulated p65 NF-κB and sirtuin-1 enhancement to reduce nucleotide-binding domain-like receptor protein 3 (NLRP3), apoptosis-associated speck-like protein (ASC), active caspase-1, and interleukin-1β, indicative of inflammasome deactivation. It also revealed an antioxidant capacity via boosting nuclear factor E2-related factor (Nrf2) enhancing glutathione through forkhead box protein O3a (FOXO3a) reduction. Additionally, CLZ triggered neuronal survival by promoting the p-content of Akt, TrkB, and CREB as well as BDNF content. A novel approach of CLZ in hindering global cerebral I/R–mediated neuropathy is firstly documented herein to forward its adjunct action via deactivating the NLRP3 inflammasome, besides enhancing Nrf2 axis, neuronal survival, and dopamine neurotransmission as well as inhibiting TRPA1 and excitotoxicity.

**Graphical abstract:**

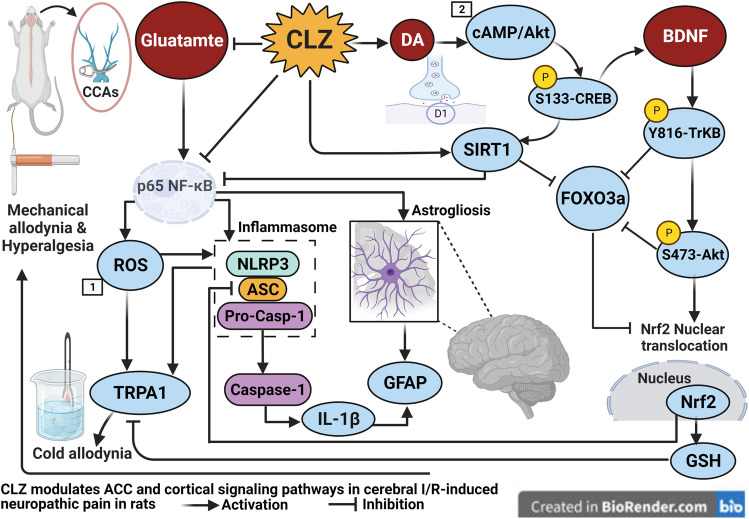

**Supplementary Information:**

The online version contains supplementary material available at 10.1007/s12035-022-03024-w.

## Introduction

Neuropathic pain is a debilitating outcome not only associated with peripheral diseases, but also as a consequence to nervous system disorders [1]. Global cerebral ischemia/reperfusion (I/R) injury, a common feature of severe ischemic decline or block in the brain blood flow proceeds in several conditions, such as cardiac arrest, and cardiovascular surgery, occurs upon restoration of blood supply after the initial ischemic insult [2]. Estimates of the prevalence of post cerebral I/R pain are around 10.6%, where central post-ischemic pain is the most frequently diagnosed neurological condition caused by central damage or dysfunction [3]. In the latter context, post-ischemic pain occurs spontaneously meanwhile responses to noxious or non-noxious stimuli are pathologically amplified following the primary insult [4]. Characteristic symptoms of neuropathic pain include tactile allodynia, viz., pain hypersensitivity to normally non-noxious stimuli upon touching and hyperalgesia, i.e., increased pain perception of noxious stimuli [5].

Cerebral I/R leads to complex maladaptive sensory neural network alterations by affecting the spinothalamic pathways [6]. It is worth pointing out that evoked responses arising from sensory inputs relay on different nuclei along somatosensory pathways [7]. Notably, sensory information is transmitted afferently to the dorsal horn of the spinal cord then to the supraspinal cord areas finally reaching the cerebral cortex through the spinothalamic tract and interpreted as pain [8]. Chiefly, anterior cingulate cortex (ACC) is the key cortical brain area in the somatosensory system which is involved in the processing of both sensory nociceptive information and the anticipation of painful stimuli [9]. This could be attributed to its very rapid excitatory transmission synaptic mechanism for nociception after tissue injury [10]. Additionally, sustained excitatory input into the ACC induces microglial activation, hence worsening detrimental insults [11]. Intensified excitatory glutamate (Glu) release and signaling of glutamatergic receptors have been crucially linked to central sensitization of cerebral cortical tissues as ACC during neuropathic pain [12].

Apart from excitotoxicity, the inflammatory process begins in the intravascular compartment immediately after arterial occlusion [13]. Interestingly, changes in the shear stress ensue the production of reactive oxygen species (ROS) that trigger several downstream cascades [14]. These include the activation of transient receptor potential ankyrin 1 (TRPA1), a pain sensor highly expressed in nociceptive neurons detecting mechanical and cold stimuli as well as different inflammatory and oxidative substances [15, 16]. TRPA1 activation in both nociceptive dorsal root ganglion and primary afferent neurons in the spinal cord is implicated in various peripheral neuropathic pain pathogenesis as sciatic nerve injury, diabetic neuropathy, and chemotherapeutic-induced peripheral neuropathy [17, 18]; nonetheless, its role in central nociception is less addressed [18]. Moreover, oxidative stress also induces inflammasome activation, where blocking nucleotide-binding domain-like receptor protein 3 (NLRP3) signaling cascade is considered a key regulator of neuroinflammation in the CNS pathologies including central post-ischemic pain [19]. Additionally, inflammasome upregulation in dorsal root ganglion neurons, sciatic nerve infiltrated peripheral macrophages, and the spinal dorsal horn astrocytes initiates peripheral pain sensitization such as complex regional pain syndrome, gouty arthritis, and chronic constriction injury of the sciatic nerve [20, 21].

Central dopamine (DA), on the other hand, enacts a major role through the dopamine 1 (D1) receptor activation in counteracting neuropathic pain. Cerebral DA mitigation induces cold allodynia by cFOS upregulated expression in Parkinson-diseased rats [22]. Besides, DA relives mechanical allodynia and hyperalgesia through initiating the production of cyclic adenosine monophosphate (cAMP) which promotes consequent degradation and inactivation of NLRP3 as reported in models of neurotoxin-induced inflammation, lipopolysaccharide-induced systemic inflammation, and monosodium urate–induced peritonitis [23]. Meanwhile, diverse survival signals, namely brain-derived neurotrophic factor (BDNF) and anti-inflammatory cascades including Sirtuin-1 (SIRT-1) and nuclear factor E2-related factor or nuclear factor (erythroid-derived 2)-like 2 (Nrf2) with forkhead box protein O3a (FOXO3a) inhibition, have been reported to protect against neuropathic pain in human and mice [24, 25].

Cilostazol (CLZ), a selective potent inhibitor of phosphodiesterase-3 (PDE-3) [26], largely possesses antiplatelet and vasodilatory characteristics that generally abate a variety of pathological conditions including I/R as assessed experimentally [27, 28]. CLZ through inhibiting PDE-3 leads to cAMP elevation [29] which effectively alleviates NLRP3 in various inflammatory models [23]. Furthermore, PDE-3 ablation in mice plays a potent role in preventing NLRP3 activation, hence reducing inflammatory cascade [30]. Given the aforementioned pieces of tentative evidence, the current study aimed to re-introduce CLZ as a possible anti-nociceptive drug in the management of post-ischemic pain disorders. This study highlights the potential anti-nociceptive signaling pathways of CLZ in an established rat model of global cerebral I/R that was for the 1st time assessed for neuropathic pain herein with special focus on the NLRP3 inflammasome.

## Material and Methods

### Animals

Adult male Wistar rats (200–250 g) obtained from the National Research Center (Cairo, Egypt) were housed in groups and maintained on a 12/12-h light/dark cycle with food and water provided ad libitum. Housing was kept at a constant room temperature (23 ± 2 °C) and humidity level (60 ± 10%). All surgical procedures were approved by the research ethics committee of the Faculty of Pharmacy, Cairo University (Cairo, Egypt; PT: 2149) in compliance with the Guide for the Care and Use of Laboratory Animals published by the US National Institutes of Health, 8th edition (NIH Publication No. 85–23, revised 2011; National Research, 2011). All efforts were made to minimize animal pain, discomfort, and suffering.

### Transient Global Cerebral Ischemia-Induced Neuropathy Model

Rats were anesthetized with thiopental sodium (50 mg/kg, i.p) [31] and rectal temperature was maintained at 37 °C via an overhead heating lamp. A ventral midline incision was made to expose both common carotid arteries (CCAs). After careful isolation from the vagus nerve and surrounding tissues, ischemia was initiated by bilateral ligation of the CCAs using non-traumatic aneurysmal clips for 45 min [31]. Thereafter, blood flow was re-instated for 48 h after suturing the incision [32] to establish the transient global cerebral ischemia-induced neuropathy model.

### Experimental Design

**Fig. 1:**
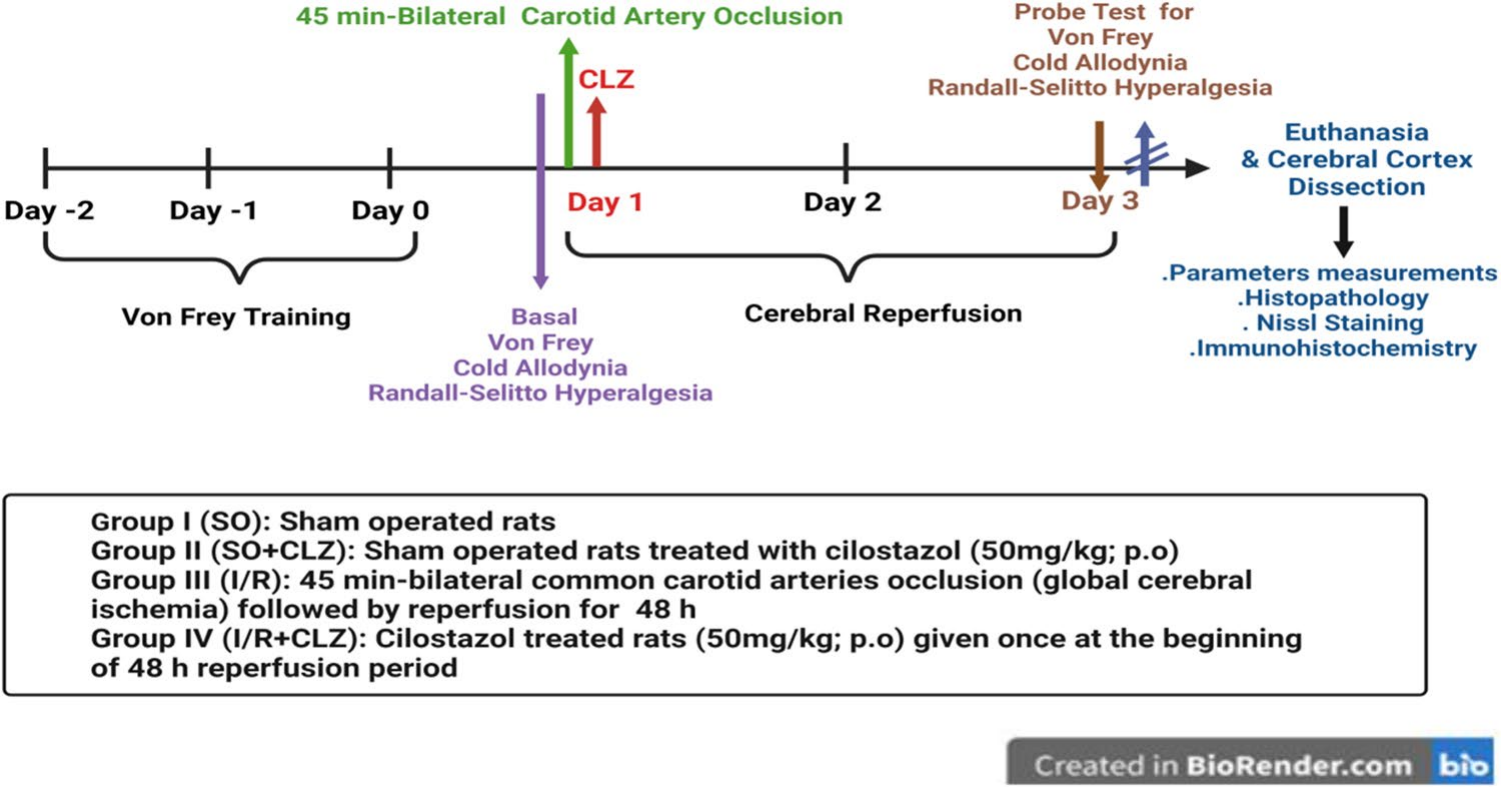
Schematic illustration of the experimental design. Rats were divided into four main groups: SO, SO + CLZ, global cerebral I/R injury, and CLZ-treated groups. Global cerebral I/R rats were given CLZ (50 mg/kg; p.o) once at the beginning of the 48 h reperfusion period. The experiment lasted for 6 days; all rats were trained on von Frey filaments for 3 days and cold allodynia for 1 day before laparotomy/operation. After 48 h of reperfusion, rats were tested for mechanical/cold allodynia and hyperalgesia using the Randell Selitto, then rats were euthanized, and either the whole brain was used for histopathological/immunohistochemical examination in the ACC area or the cerebral cortex was isolated for further immuno-/biochemical analysis. CLZ, cilostazol; I/R, ischemia/reperfusion; SO, sham operation. Created with BioRender.com

As depicted in Fig. 1, 36 rats were randomly allocated into four groups (*n* = 9) by a technical assistant who was not involved in the analysis, and all rats were subjected to behavioral testing [33]. Group size was based on a power analysis (power = 0.8, *α* = 0.05) using effect sizes previously determined by [34]. Group one rats served as sham-operated (SO) in which both CCAs were exposed without occlusion, whereas animals in the remaining two groups were subjected to 45 min ischemia followed by a 48 h perfusion period to serve as either as (1) cerebral I/R group or (2) I/R + CLZ (Sigma-Aldrich, MO, USA; 50 mg/kg; p.o; suspended in 1% Tween 80; [27]) group, where the drug was given once at the beginning of the 48 h reperfusion period. Notably, animals in both the − ve and + ve control groups received the vehicle at the same timeline of the treatment (Fig. 1). Each group was further subdivided into two subsets that were utilized as follows: (i) in the first set (*n* = 6 rat per group), both cortices were used for ELISA estimation, and (ii) in the second set (*n* = 3 rat per group), the brain was dissected into 2 hemispheres where the left one was used for Western blot and the right was fixed in 10% phosphate-buffered formalin for histopathological and immunohistochemical examination. During the analysis of these measurements, the investigators were blinded to sample identity, and sample coding and decoding were performed by an independent experimenter.

### Behavioral Tests

To acclimatize the rats to the testing techniques and to accomplish a steady baseline record, the von Fery procedure was daily performed for 3 days before surgery after a 30 min habituation period in meshed floor cages [35], whereas basal cold allodynia [36] and hyperalgesia [37] assessments were done the day before cerebral I/R induction. During the animal’s light cycle and 48 h after the start of the reperfusion period, behavioral tests were conducted with a minimum of 30 min [38] between each for all animals utilized in this study (Fig. 1).

#### von Frey Test for Mechanical Allodynia

Rats were habituated for 30 min in a custom-made elevated wire mesh floor platform surrounded by a clear glass chamber (10 × 10 × 15 cm). von Frey single monofilaments with gradually increasing strengths (forces: 6, 8, 10, 15, 26, 60, 100, 180, and 360 g; probe equivalent evaluator size: 4.74, 4.93, 5.07, 5.18, 5.46, 5.88, 6.1, 6.45, and 6.65; Ugo Basile, VA, Italy) were applied to the paw plantar aspect. Each filament was directed vertically with adequate force through the wire mesh towards the sub-planter region of the paw till bent; a response ranked either 0 indicative of no response or 1 which presents a brisk or flinching withdrawal was recorded for each trial. The procedure was repeated five times on each hind paw at 30 sec intervals. According to the “percent response” method [39], the 60% paw withdrawal threshold (PWT), presenting tactile allodynia, is calculated from the lowest force that caused at least 3 withdrawals out of the 5 consecutive applications [40].

#### Cold Allodynia Test

Cold allodynia was determined in accordance with [36]. The paw withdrawal latency (PWL; sec) of dipping the hindlimb in iced cool water (4 ± 1 °C) was recorded.

#### Randall-Selitto Hyperalgesia Test

Mechanical hyperalgesia was quantified using the method of Randall-Selitto [37]. Briefly, using an Ugo Basile analgesimeter with the probe tip of 1-mm diameter, an increasing pressure was applied to the dorsal surface of the right hind paw. The nociceptive PWT was defined as the force (g) at which the animal attempted to withdraw its paw; a maximal cut-off of 750 g was used to prevent tissue damage.

### Sampling

After behavioral testing, animals were euthanized, and the cerebral cortexes were dissected on dry ice, where both cortices of animals in the 1st experimental subset were homogenized in ice-cold phosphate buffer saline (PBS; pH 7.4) for ELISA parameters assessments and the left cortex of rats in the 2nd subset was homogenized in RIPA buffer provided with protease inhibitor for Western blot technique. All homogenates were kept in aliquots and stored at − 80 °C until use. For cortical nuclear p65 NF-κB and Nrf2 content determination, the cortex homogenates were dispersed in an ice-cold lysis buffer. Afterwards, the nuclear extract was prepared using a commercially available nuclear extraction kit (Abcam, MA, USA, Cat#AB113474) according to the manufacturer’s instructions. Of note, all parameters were normalized to the protein content measured according to the Bradford assay [41].

### Cortical Inflammatory and ROS Biomarkers, DA, Glu, TRPA1, and BDNF, Phospho-Akt/-CREB/-TrkB as well as Nrf2

Cortical p65 NF-κB (MyBioSource, CA, USA, Cat#MBS015549), NLRP3 (MyBioSource, CA, USA, Cat#MBS2706815), apoptosis-associated speck-like protein (ASC; MyBioSource, CA, USA, Cat#MBS7215885), interleukin-1 beta (IL-1β; MyBioSource, CA, USA, Cat# MBS702717), DA (CUSABIO, TX, USA, Cat#CSB-E08660r), Glu (Arigo Biolab, Hsinchu, Taiwan, Cat#ARG80453), SIRT-1 (FineTest, Wuhan, PRC, Cat#ER1338), pS133-cAMP responsive element binding (CREB; Duoset, MN, USA, Cat#DYC2510-2), pS473-protein kinase B (Akt/PKB; RayBio, GA, USA, Cat#PEL-AKT-S473-T), pY816-receptor tropomyosin receptor kinase B (TrkB; Assay solution, MA, USA, Cat#AYQ-E10348), BDNF (Elab, Wuhan, PRC, Cat#E-EL-R1235), TRPA1 (Abclonal, MA, USA, Cat#RK04268), glutathione (GSH; Assay Genie, Dublin, Ireland, Cat#RTEB1811), and Nrf2 (MyBioSource, CA, USA, Cat#MBS752046( were quantified using the commercially available ELISA kits. All the procedures were performed according to the manufacturer’s instructions.

### Western Blot Analysis for FOXO3a and Cleaved Caspase-1 Protein Expressions

Proteins were extracted from cortical tissues using the Bio-Rad Protein Assay kit (Bio-Rad, CA, USA, Cat#500002); equal amounts of proteins were loaded onto 8% sodium dodecyl sulfate–polyacrylamide gels and separated by electrophoresis (Bio-Rad) according to their molecular weights: total caspase-1 (45 kDa), cleaved caspase-1 (20 kDa), and FOXO3a (90 kDa). Following electrophoresis, proteins were transferred to nitrocellulose membranes (Amersham Bioscience, NJ, USA) using a semidry transfer apparatus (Bio-Rad). Membranes were blocked with 5% bovine serum albumin in Tris-buffered saline containing 0.05% Tween 20 (TBST) at 4 °C overnight. Afterwards, membranes were incubated with diluted primary antibodies against total caspase-1 (1:500; Thermo Fisher Scientific, Cat#PA5-86936), cleaved caspase-1 (1:500; Thermo Fisher Scientific, Cat#PA5-99390), FOXO3a (1:1000; Thermo Fisher Scientific, Cat#PA5-27145), and β-actin (1:500; Thermo Fisher Scientific, Cat#PA5-85271) for 1 h at room temperature with constant shaking. The membranes were washed and incubated with horseradish peroxidase-conjugated goat anti-mouse immunoglobulin (1:2000; Dianova, Hamburg, Germany, Cat#DAB-87584) for 1 h at room temperature. Finally, the band intensity was analyzed using a ChemiDoc™ imaging system with Image Lab™ software version 5.1 (Bio-Rad). Results were presented in arbitrary units after normalization to levels of the β-actin protein.

### Microscopic Analysis

All methods of tissue preparation and staining are outlined by [42] and assessments were performed blinded. Following a 48 h reperfusion period, brains were separated and immediately fixed in 10% phosphate-buffered formalin for 72 h. Consequently, brains were embedded in paraffin, and 5-μm-thick tissue sections were made. Six random non-overlapping fields were scanned and analyzed from all animals using a light microscope (Leica Microsystems GmbH, Wetzlar, Germany).

#### Histopathological H&E Staining and Injury Grading of the ACC Area

Brain sections were stained with hematoxylin and eosin (H&E) to determine microscopic alterations as well as to quantify the individual ACC damage scores. The histopathological lesions were evaluated using a previous scoring system [43], where the scores designated with a 4-point scale: 0, 1, 2, and 3 indicate no changes, mild (changes < 30%), moderate (changes 30–50%), and severe (changes > 50%) alterations, respectively. All histopathological processing and assessment of specimens were performed by an experienced observer unaware of the identity of the sample being examined to avoid any bias.

#### Nissl Staining to Assess ACC Neuronal Viability

Toluidine blue was used for the detection of intact neurons in the ACC area; neurons with visible nuclei, clear nucleoplasm, and distinctive nucleolus were counted and the mean number of intact viable Nissl-stained ACC neurons per field was calculated.

#### GFAP Immunoreactivity in the ACC Area

Avidin–biotin complex (ABC) technique was used for immunohistochemical detection of glial fibrillary acidic protein (GFAP) within the ACC. Dewaxed and dehydrated sections were incubated overnight at 4 °C with rat anti-GFAP antibody (Thermo Fischer Scientific, Cat#13–0300; 1:200). Visualization was done using commercial ABC (Santa Cruz Biotech, CA, USA) for 30 min. Afterwards, sections were exposed to 3,3′-diaminobenzidine tetrahydrochloride (DAB) as the chromogen and then counterstained with hematoxylin. Positive GFAP reaction was detected as the brown coloration of the astrocytes including their bodies and processes and GFAP percentage of immunoreactivity was recorded.

### Statistical Analysis

All data were checked for normality as well as homogeneity of variance prior to one-way analysis of variance (ANOVA) analyses using Kolmogorov–Smirnov and Bartlett’s tests, respectively. Data sets that met the assumptions for parametric analysis were analyzed using one-way ANOVA followed by Tukey’s multiple comparisons test and were expressed as mean ± SD. Two-way repeated-measures ANOVA followed by Bonferroni’s multiple comparisons test was performed for the von Frey, cold allodynia, and hyperalgesia (pre-post operation). Results of analysis and data were expressed as mean ± SD. For the histopathological scores, statistical analysis was carried out using the non-parametric Mann–Whitney test to compare every two groups, and data were expressed as minimum, maximum, median, and the first and third quartiles. A probability level of less than 0.05 was accepted as statistically significant. Statistical analysis was performed using GraphPad Prism 9.4.0 (GraphPad Software Inc., CA, USA) software.

## Results

It is noteworthy that the statistical analysis of SO vs SO + CLZ groups’ results showed no significant difference in all measured parameters. Hence, all parameters’ results were done as compared to SO-subjected rats only.

### CLZ Alleviates Global Cerebral I/R-Induced Punctate Mechanical Allodynia/Static Hyperalgesia, Cold Allodynia, and ACC Damage in Rats

**Fig. 2:**
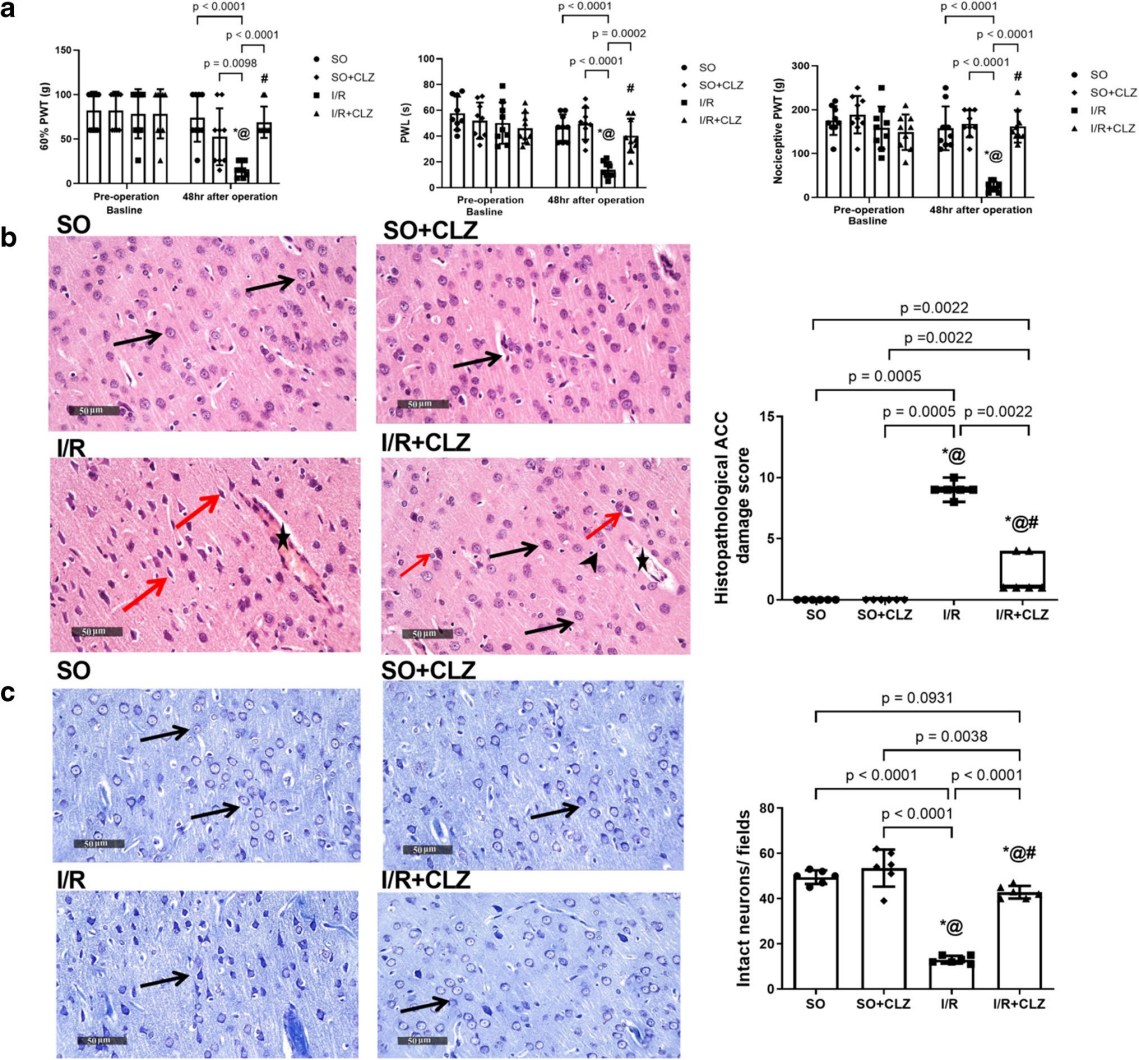
Effect of CLZ on **a** mechanosensory PWT, PWL to 4 °C cold water stimulus, and nociceptive PWT as well as ACC area; **b** histopathological changes; and **c** intact neuronal count in rats subjected to global cerebral I/R injury. **b** Photomicrograph of H&E stained ACC (× 400) sections from SO group showed apparent intact neurons with minimal glial cells infiltrates and intact intercellular matrix; SO + CLZ group showed normal appearance with intact neurons; I/R group showed diffuse neuronal damage and many figures of shrunken neurons, degenerative neuronal records with nuclear pyknosis, moderate perineuronal edema and higher records of glial cells infiltrates including astrocytic infiltrates (arrowhead), and moderate congested cerebral blood vessels (star); CLZ group showed apparent intact, protected neurons (black arrow) with occasional sporadic records of degenerative neuronal changes (red arrow). Dot boxplot chart presents the collective histopathological ACC damage score. **c** Photomicrograph of Nissl-stained ACC (× 400) sections from SO group showed intact neurons (black arrow); I/R group showed damaged neurons (black arrow); CLZ group showed intact neurons (black arrow). The bar chart represents the quantitative analysis of intact neurons number. Non-parametric data (average of 6 fields of *n* = 3 rats per group for H&E stain) are drawn as dot boxplots displaying minimum, maximum, median, and the first and third quartiles that were analyzed by the Mann–Whitney test to compare every two groups, whereas for parametric data (*n* = 9 rats per group for allodynia and hyperalgesia), each bar with vertical line represents mean ± SD that were analyzed using two-way repeated-measures ANOVA followed by Bonferroni’s post hoc test; for average of 6 fields (*n* = 3 rats per group for Nissl stain), each bar with vertical line represents mean ± SD that were analyzed using one-way ANOVA followed by Tukey’s post hoc test; *p* < 0.05, * vs SO, @ vs SO + CLZ, # vs I/R. ACC, anterior cingulate cortex; CLZ, cilostazol; H&E, hematoxylin and eosin; I/R, ischemia/reperfusion; PWT, paw withdrawal threshold; PWL, paw withdrawal latency; SO, sham operation

Rats in the global cerebral I/R group exhibited significant neuropathic pain including both mechanical hyperalgesia and mechanical/cold allodynia. This was manifested by marked declines in withdrawal latency of the hind paw in response to the von Frey filament test indicative of low-threshold mechanoreceptors stimulation/nociceptive sensitization by 22% and mechanical hyperalgesia assessed by Randall-Selitto test by 15% versus SO (Fig. 2a). Repeated-measures two-way ANOVA analysis of mechanical allodynia test showed significance between groups (*F*(3,32) = 5.265, *p* = 0.0046), a significant effect of time (pre-post operation) (*F*(1,32) = 27.08, *p* < 0.0001), and a significant interaction between time and groups (*F*(3,32) = 5.727, *p* < 0.0030). Repeated-measures two-way ANOVA analysis of hyperalgesia revealed significance between groups (*F*(3,32) = 16.01, *p* = 0.0001), significant effect of time (pre-post operation) (*F*(1,32) = 22.57, *p* < 0.0001), and a significant interaction between time and groups (*F*(3,32) = 14.83, *p* < 0.0001). Additionally, I/R rats showed a noticeable reduction in the PWL by 30% in response to cold stimulation, quantifying thermal allodynia, relative to SO rats (Fig. 2a). Repeated-measures two-way ANOVA analysis of cold allodynia showed significance between groups (*F*(3,32) = 7.342, *p* = 0.0007), significant effect of time (pre-post operation) (*F*(1,32) = 35.53, *p* < 0.0001), and a significant interaction between time and groups (*F*(3,32) = 10.93, *p* < 0.0001). CLZ post-treatment proved significant antihyperalgesic activity where it abated both mechanical and cold allodynia as well as static hyperalgesia induced by the transient global ischemic insult to reach SO values. Meanwhile, there was no significant difference at the baseline between groups in all aforementioned behavioral tests. In the ACC area, I/R produced diffuse neuronal damage represented as pyknosis (Fig. 2b) with reduced intact neurons by 26% (*F*(3,20) = 93.48, *p* < 0.0001, Fig. 2c) associated with higher number of glial cell infiltrates (Fig. 2b), besides the presence of edema and congested blood vessels (Fig. 2b) versus SO rats exhibiting normal histological organization (Fig. 2b) with apparently intact densely packed neurons (Fig. 2b). Nonetheless, CLZ post-treatment amended the I/R-induced cortical damage as noticed by occasional sporadic degenerative neuronal changes, reduced number of glia, and mildly congested blood vessels (Fig. 2b). This was reflected by the partial improving effect of CLZ depicted from both the reduction in the sum of individual scores for neurodegeneration, edema, and glial cell infiltration by 22% of the insult value (*p* = 0.0022) (Fig. 2b) and the promotion of neuronal survival by 3.3 folds versus I/R (*F*(3,20) = 93.48, *p* < 0.0001) in the ACC area (Fig. 2c).

### CLZ Modulates Cortical TRPA1 Content as well as Glu and DA Neurotransmission Following Global Cerebral I/R Injury in Rats

**Fig. 3:**
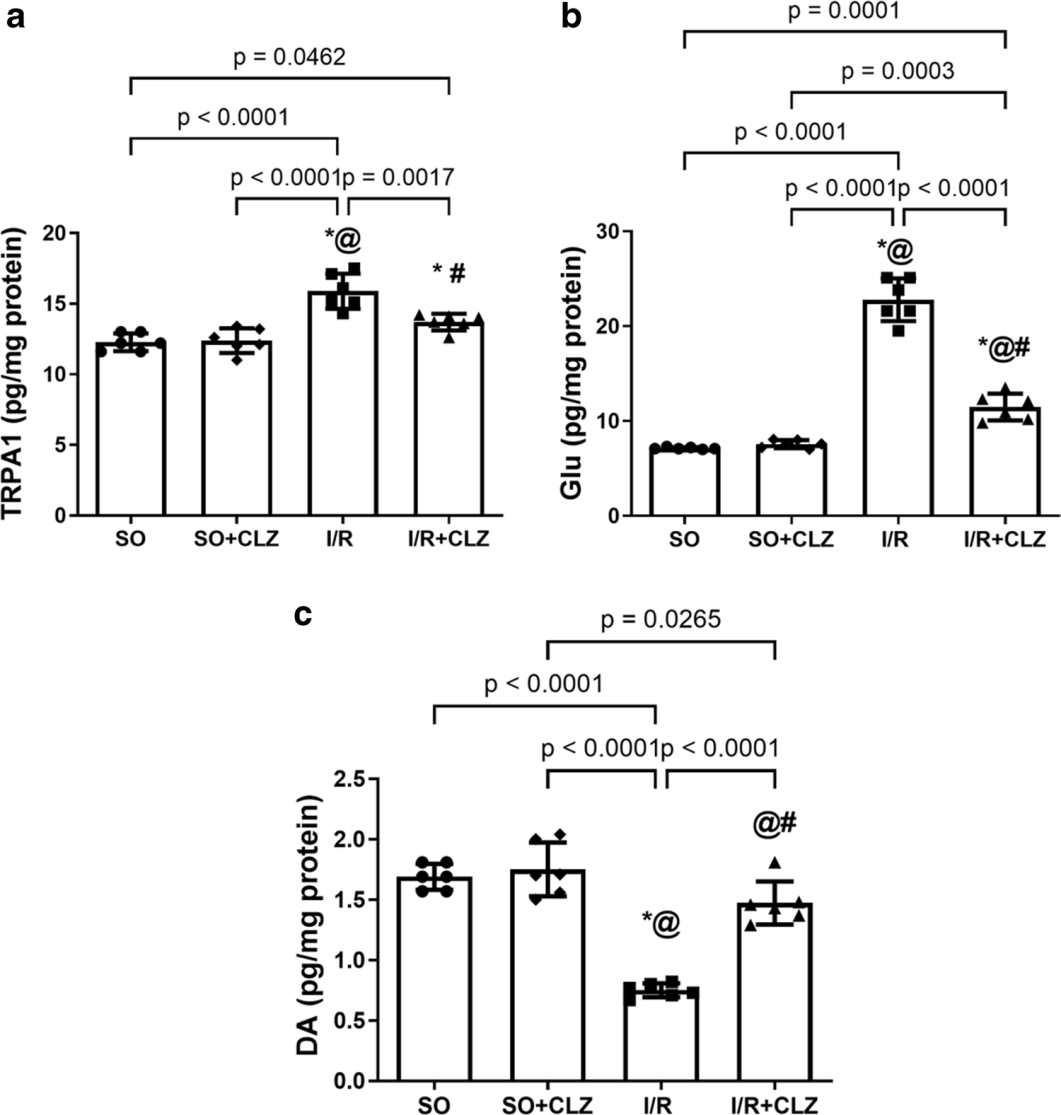
Effect of CLZ on cortical content of **a** TRPA1, **b** DA, and **c** Glu in rats subjected to global cerebral I/R injury. Each bar with a vertical line represents mean ± SD (*n* = 6 rats per group) and was analyzed using one-way ANOVA followed by Tukey’s post hoc test; *p* < 0.05, * vs SO, @ vs SO + CLZ, # vs I/R. CLZ, cilostazol; DA, dopamine; Glu, glutamate; I/R, ischemia/reperfusion; SO, sham operation; TRPA1, transient receptor potential ankyrin 1

Interplay of central factors contributes to neuropathic pain sensation. Cerebral I/R injury raised the cortical content of TRPA1 by 1.3 fold (*F*(3,20) = 22.14, *p* < 0.0001, Fig. 3a) and Glu by 3.2 folds (*F*(3,20) = 176.4, *p* < 0.0001, Fig. 3b) with the reduction in that of DA by 44% (*F*(3,20) = 52.57, *p* < 0.0001, Fig. 3c) compared to the SO group. On the contrary, CLZ post-treatment partially reverted such effects where TRPA1 dropped by 86% (*F*(3,20) = 22.14, *p* < 0.0001) and Glu was almost halved by 50% (*F*(3,20) = 176.4, *p* < 0.0001), effects that aligned a twofold (*F*(3,20) = 52.57, *p* < 0.0001) increment in DA relative to the insult.

### CLZ Abates Global Cerebral I/R-Induced NF-κB-Mediated Canonical NLRP3 Inflammasome Activation in the Rat Cerebral Cortex

**Fig. 4:**
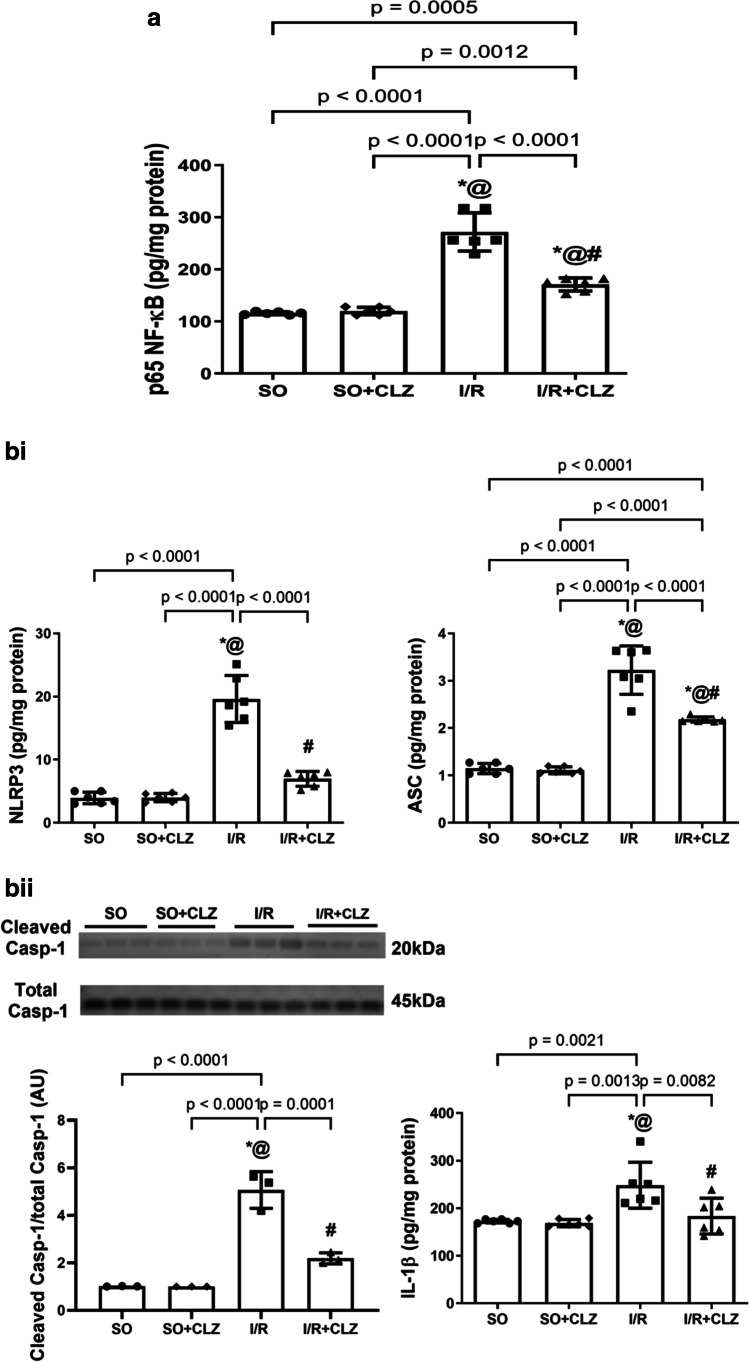
Effect of CLZ on cortical content of **a** p65 NF-ĸB nuclear fraction; **bi–bii** NLRP3, ASC, and IL-1β, and protein expression of cleaved caspase-1/total caspase-1 ratio in rats subjected to global cerebral I/R injury. Each bar with a vertical line represents mean ± SD (*n* = 6 rats per group) and was analyzed using one-way ANOVA followed by Tukey’s post hoc test; *p* < 0.05, * vs SO, @ vs SO + CLZ, # vs I/R. ASC, apoptosis-associated speck-like protein; CLZ, cilostazol; Casp-1, caspase-1; IL-1β, interleukin-1beta; I/R, ischemia/reperfusion; p65 NF-ĸB, nuclear factor kappa B; NLRP3, nucleotide-binding domain-like receptor protein 3; SO, sham operation

Following the activation of TRPA1 and Glu receptor, while inhibiting the DA pathway, cerebral I/R increased p65 NF-κB to 2.3 folds (*F*(3,20) = 81.29, *p* < 0.0001, Fig. 4a) versus SO. These anomalies triggered NLRP3 inflammasome assembly/activation indicated by the increased content of NLRP3 by 5 folds (*F*(3,20) = 80.75, *p* < 0.0001, Fig. 4bi) and ASC by 2.8 folds (*F*(3,20) = 86.83, *p* < 0.0001, Fig. 4bi). The inflammasome downstream neuroinflammatory cascade was enhanced as depicted by the increment in the IL-1β content by 1.4 folds (*F*(3,20) = 8.697, *p* = 0.0007, Fig. 4bii) upon activation by cleaved caspase-1 which protein expression relative to total caspase-1 augmented by 5 folds (*F*(3,8) = 68.19, *p* < 0.0001, Fig. 4b; uncropped blot of 4bii, Supplementary Fig. S1a) was boosted above SO. On the other hand, CLZ post-treatment amended such verities to downregulate p65 NF-κB by 63% (*F*(3,20) = 81.29, *p* < 0.0001) with decrements in NLRP3 by 36% (*F*(3,20) = 80.75, *p* < 0.0001), ASC by 67% (*F*(3,20) = 86.83, *p* < 0.0001), and cleaved caspase-1/total caspase-1 ratio by 43% (*F*(3,8) = 68.19, *p* < 0.0001) and the normalization of the IL-1β content by 74% (*F*(3,20) = 8.697, *p* = 0.0007) compared to the insult.

### CLZ Activates Cortical BDNF/TrkB/Akt/CREB Hub and Following the Global Cerebral I/R Insult in Rats

**Fig. 5:**
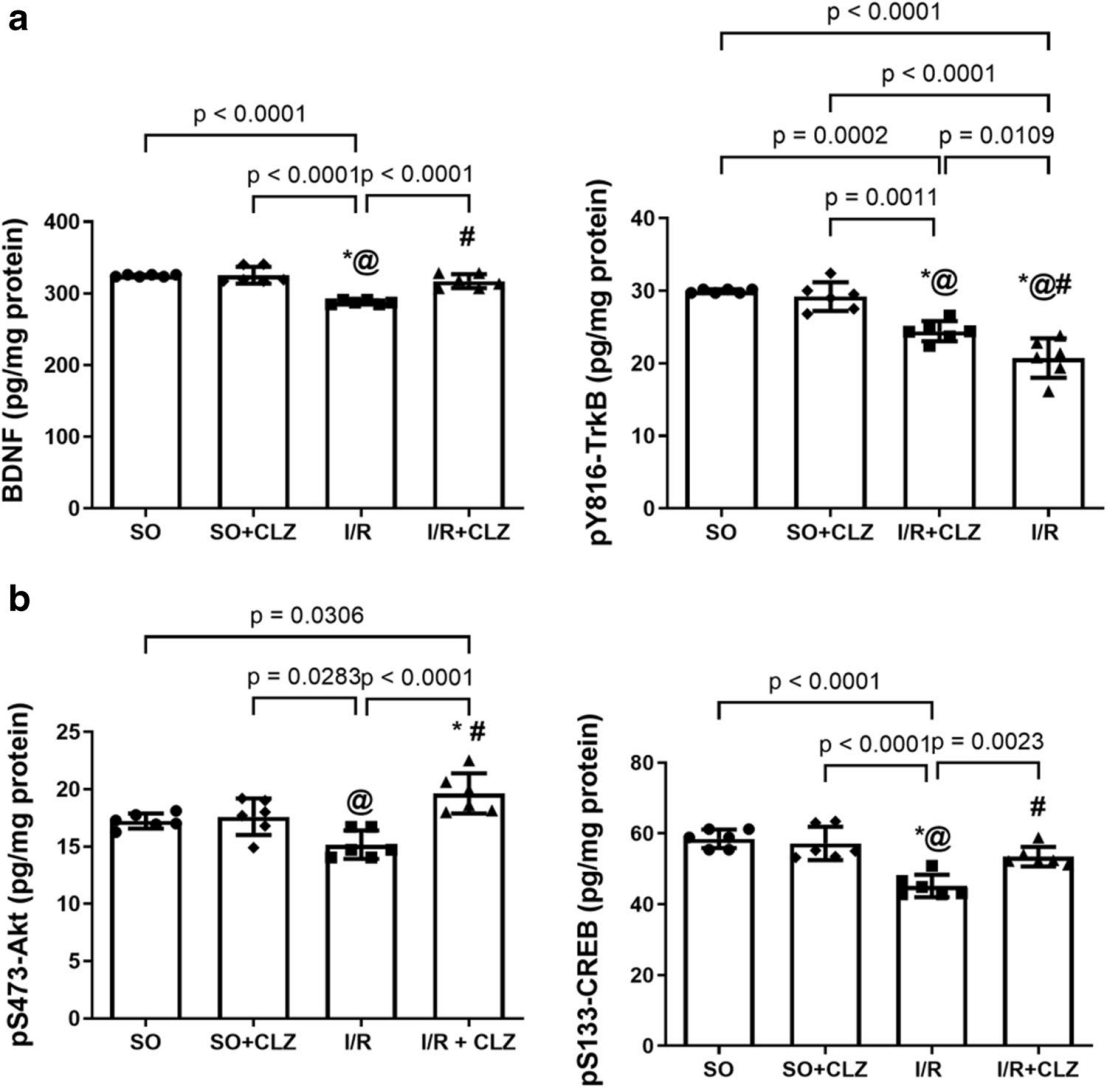
Effect of CLZ on cortical content of **a** BDNF and p-TrkB and **b** p-Akt and p-CREB in rats subjected to global cerebral I/R injury. Each bar with a vertical line represents mean ± SD (*n* = 6 rats per group) and was analyzed using one-way ANOVA followed by Tukey’s post hoc test; *p* < 0.05, * vs SO, @ vs SO + CLZ, # vs I/R. Akt, serine/threonine protein kinase B; BDNF, brain-derived neurotrophic factor; CLZ, cilostazol; CREB, cAMP response element-binding protein; I/R, ischemia/reperfusion; SO, sham operation; TrkB, tropomyosin receptor kinase B

The BDNF/TrkB/Akt/CREB axis promotes neuronal cell survival and is an important signaling neuroprotective pathway against cerebral I/R injury. Compared to SO, I/R suppressed the cortical BDNF content by 89% (*F*(3,20) = 31.88, *p* < 0.0001, Fig. 5a) and p-content of TrkB by 69% (*F*(3,20) = 33.78, *p* < 0.0001, Fig. 5a), Akt by 88% (*F*(3,20) = 10.70, *p* = 0.0002, Fig. 5b) and CREB by 77% (*F*(3,20) = 18.49, *p* < 0.0001, Fig. 5b) versus SO. On the other hand, the post-treatment with CLZ in part counteracted the I/R effects to different extents on all the aforementioned marker contents and normalized BDNF.

### CLZ Boosts Cortical SIRT-1 and GSH viaModulating the FOXO3a/Nrf2 Axis in Global Cerebral I/R Rats

**Fig. 6:**
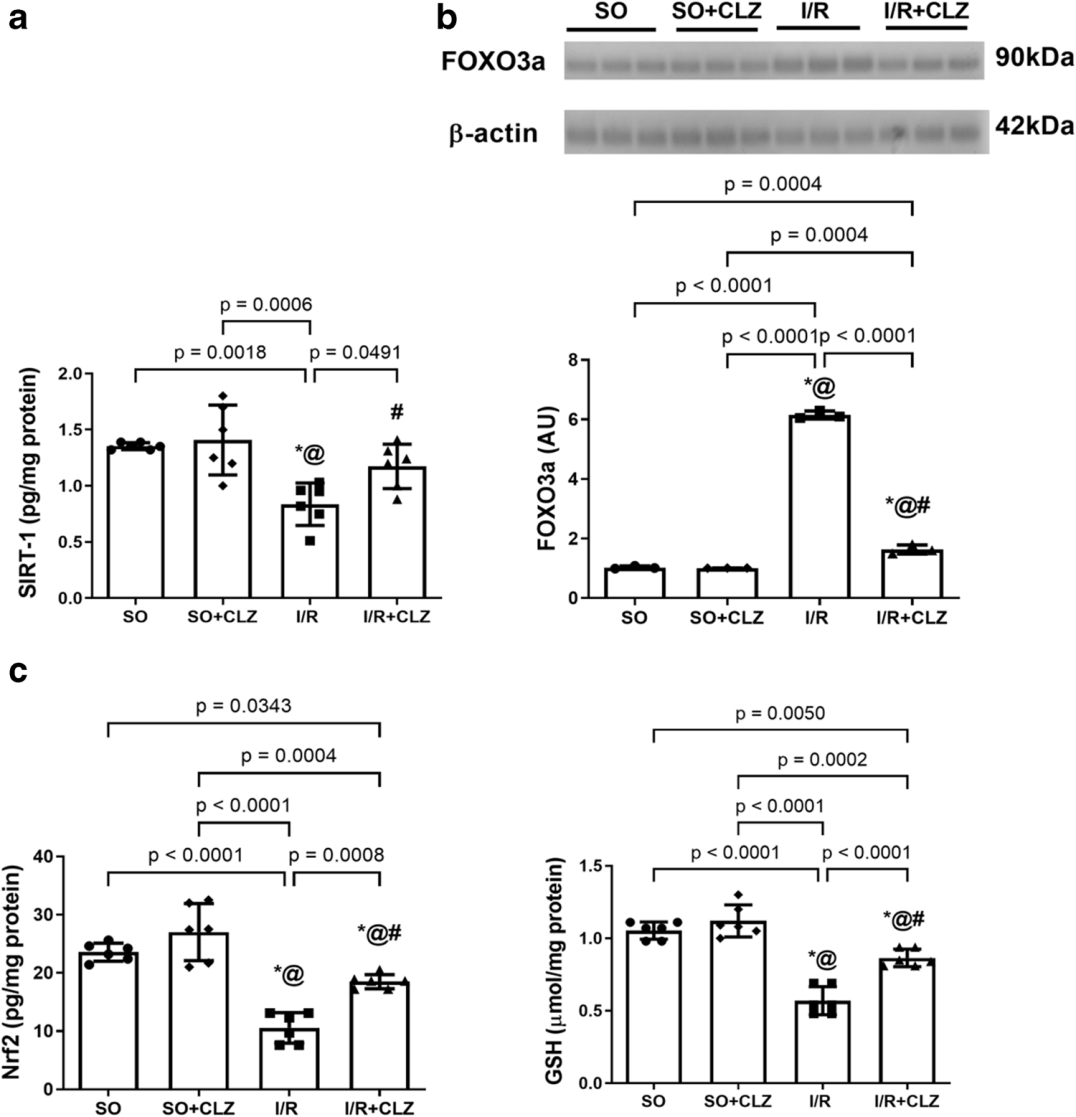
Effect of CLZ on cortical content of **a** SIRT-1, **b** protein expression of FOXO3a, **c** content of Nrf2 nuclear fraction and GSH in rats subjected to global cerebral I/R injury. Each bar with a vertical line represents mean ± SD (*n* = 6 rats per group) and was analyzed using one-way ANOVA followed by Tukey’s post hoc test; *p* < 0.05, * vs SO, @ vs SO + CLZ, # vs I/R. CLZ, cilostazol; FOXO3a, forkhead box O3a; GSH, glutathione; I/R, ischemia/reperfusion; Nrf2, nuclear factor erythroid 2-related factor 2; SIRT-1, Sirtuin-1; SO, sham operation

FOXO3a/Nrf2 axis is known to be involved in the regulation of oxidative stress. Cerebral I/R reduced cortical SIRT-1 content by 62% (*F*(3,20) = 9.245, *p* = 0.0005, Fig. 6a). Hence, the cortical FOXO3a protein expression was increased to 6 folds (*F*(3,8) = 1714, *p* < 0.0001, Fig. 6b; uncropped blot of 6b, Supplementary Fig. S1b) with a downregulated Nrf2 by 45% (*F*(3,20) = 35.35, *p* < 0.0001, Fig. 6c) and diminished GSH content by 54% (*F*(3,20) = 50.81, *p* < 0.0001, Fig. 6c) in I/R rats compared to SO. Notably, CLZ administration enhanced SIRT-1 by 1.3 folds (*F*(2,15) = 16.13, *p* = 0.0173) and replenished the major antioxidant pool by 1.5 folds (*F*(3,20) = 50.81, *p* < 0.0001) through reducing FOXO3a by 27% (*F*(3,8) = 1714, *p* < 0.0001) and Nrf2 by 1.8 folds (*F*(3,20) = 35.35, *p* < 0.0001) compared to the I/R insult.

### CLZ Suppresses Astrocyte Activation in the ACC Area of Global Cerebral I/R Rats

**Fig. 7:**
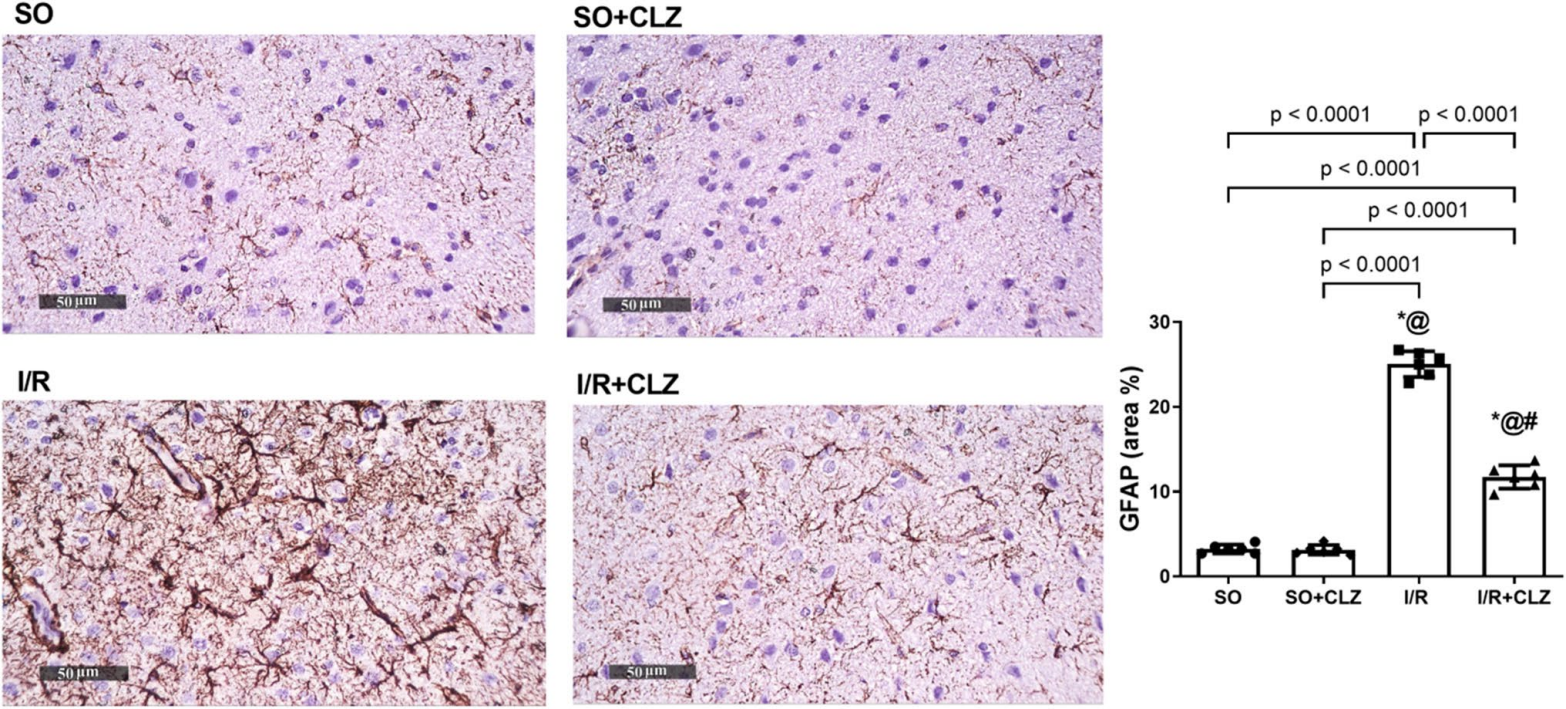
Effect of CLZ on global cerebral I/R-induced GFAP immunoreactivity in the ACC area. SO group and SO + CLZ group showed normal GFAP immunostaining, I/R group induced GFAP immunoreactive astrocytes, and CLZ treatment preserved GFAP%. Immunostaining of GFAP (× 400). The bar chart represents the quantitative analysis of GFAP immunoreactive astrocytes (% area). Each bar with a vertical line represents mean ± SD (average of 6 fields of *n* = 3 rats per group) and was analyzed using one-way ANOVA followed by Tukey’s post hoc test; *p* < 0.05, * vs SO, @ vs SO + CLZ, # vs I/R. ACC, anterior cingulate cortex; CLZ, cilostazol; GFAP, glial fibrillary acidic protein; I/R, ischemia/reperfusion; SO, sham operation

Marked astroglial activation was observed as intense GFAP immunoreactivity by 7.7 (*F*(3,20) = 535.0, *p* < 0.0001, Fig. 7) in the ACC area of I/R rats as compared to its weak expression in the SO counterparts (Fig. 7). The therapeutic benefit of CLZ was highlighted as mild immune-reactive astrocytes with lightly stained processes indicative of astroglial deactivation (Fig. 7).

## Discussion

**Fig. 8:**
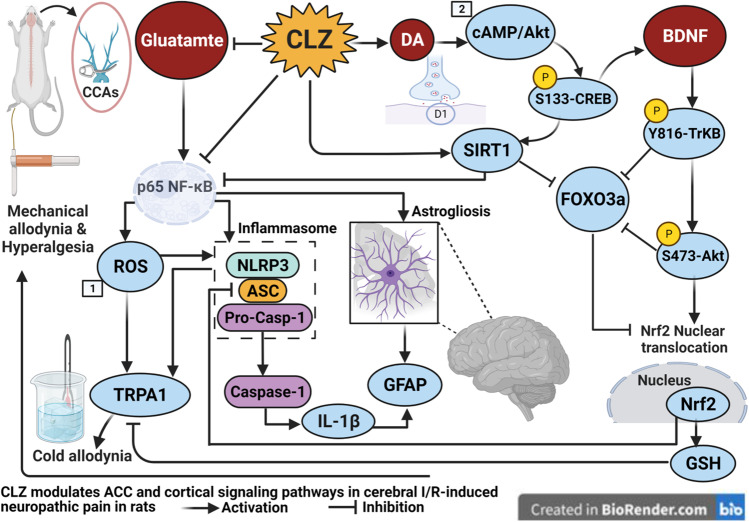
CLZ mitigates global cerebral I/R–mediated neuropathy both allodynia and hyperalgesia via deactivating the NLRP3 inflammasome. This is achieved by inhibiting both TRPA1 and glutamate signals with the activation of the neuronal Akt survival pathway linked to dopamine and BDNF, besides enhancing the Nrf2 axis. Akt, serine/threonine protein kinase B; ASC, apoptosis-associated speck-like protein; BDNF, brain-derived neurotrophic factor; Casp-1, caspase-1; CLZ, cilostazol; CREB, cAMP response element-binding protein; FOXO3a, forkhead box O3a; Glu, glutamate; GSH, glutathione; IL-1β, interleukin-1beta; I/R, ischemia/reperfusion; p65 NF-ĸB, nuclear factor kappa B; NLRP3, nucleotide-binding domain-like receptor protein 3; Nrf2, nuclear factor erythroid 2-related factor 2; SIRT-1, Sirtuin-1; TrkB, tropomyosin receptor kinase B; SO, sham operation; 1 [44]; 2 [45]; Created with BioRender.com

The current study is the 1st, to the authors’ knowledge, to verify neuropathic pain both allodynia and hyperalgesia simultaneous to transient global cerebral ischemia insult resembling post-stoke pain in patients. Herein, we used the von Frey test for mechanical allodynia, cold allodynia test, and Randall-Selitto hyperalgesia test to confirm impaired functional behavior associating with the cerebral I/R paradigm. Our study also provides substantial evidence for the ability of CLZ to dampen neuropathic pain sensation and the intensified response to non-painful mechanical and cold stimuli consequent to global cerebral I/R injury. Such phenomenon was manifested as structural amendment of the ACC area which is crucial for pain acquisition hence improving functional analgesia. In this context, CLZ mitigated the formation of NLRP3, ASC, cleaved caspase 1, and IL-1β, indicative of the deactivation of the NLRP3 inflammasome that plays a key role in central post-ischemic pain. By virtue of its pleiotropic effects, CLZ blunted the excitatory ion channel TRPA1 and neurotransmitter Glu content in the cerebral cortex to prohibit the NLRP3 inflammasome assembly and activation via the downregulation of p65 NF-κB decreasing IL-1β-mediated neuroinflammation that contributes to allodynia/hyperalgesia. Additionally, CLZ deactivated the NLRP3 inflammasome by increasing DA which promotes pain plasticity and by stalling FOXO3a to enhance the SIRT-1/Nrf2 cue with the consequent replenishment of GSH to decrease therefore the ROS burden. Meanwhile, CLZ enhanced neuronal survival by advancing the Akt/CREB/BDNF/TrkB cascade which also activates Nrf2 and reduces inflammasome assembly. Besides reduced astrogliosis, the antioxidant effect of CLZ plays a further role in the NLRP3 inflammasome and TRPA1 deactivation to halt the vicious cycle of the inflammasome activation and hence the exaggerated painful responses (Fig. 8).

In the current investigation, CLZ deactivation of the NLRP3 inflammasome is the underpins of the contracted central neuropathic pain perception verified herein by a series of behavioral tests assessing both mechanical and cold allodynia, as well as hyperalgesia using the Randal Sellito analgesy-meter consequent to cerebral transient ischemic insult. Notably, Thacheril et al. [46] reported that the drug ameliorated mechanical hyperalgesia/allodynia provoked by vincristine in mice. However, to the authors’ knowledge, this is the first report documenting the capacity of CLZ post-treatment to effectively abrogate the crucial NLRP3 inflammasome mechanistic pathway triggered by glutamatergic transmission associated with central pain sensation. Ample evidence implicate NLRP3 inflammasome as a molecular caspase-1-activating platform that triggers the processing of pro-IL-1β in a two steps process, namely priming, which upregulates the expression of inflammasome components, preceding the activation step [47]. The priming step is initiated by the recognition of various triggers, viz., NLRP3 activators, which leads to the activation/transcription of NF-κB [48]. Firstly, the modulation of Glu signaling is embroiled in neuropathic pain amid changes and reorganization of the nociceptive network [12]. Indeed, mechanical/cold allodynia as well as hyperalgesia have been previously conferred by several investigators [9, 49] declaring the potential of Glu in central sensitization associated with chronic pain. In mechanical allodynia/hyperalgesia, Glu acts via the continuous hyperactivation of *N*-methyl-d-aspartate receptor (NMDAR) in the ACC area [11] and the metabotropic glutamate receptors contributing to pain sensation/transmission [12] with the consequent activation of NF-κB that transcribes IL-1β involved in neuropathic pain [50]. This pro-inflammatory cytokine is produced following the cleavage of pro-IL-1β through the NLRP3 inflammasome assembly and activation [47]. Indeed, Zhang et al. [51] characterized the NLRP3 inflammasome as a target for pain therapy with central participation for IL-1β in higher brain regions including prefrontal cortex after sciatic nerve injury and spinal nerve ligation. The current study extends the former data to advocate a role for the involvement of the cerebral cortical NLRP3 inflammasome assembly/activation in the central response to neuropathic pain after the transient cerebral ischemic insult. In fact, the NLRP3 activation causes cerebral cortical perturbations to decrease descending projection fiber to the thalamus reducing thus GABAergic transmission associated with the enhancement of ventral basal neuronal excitability to ultimately endorse central post-ischemic pain [19]; this network alteration by NLRP3 inflammasome activation further substantiates the current data in the global cerebral I/R model. In support, inflammasome activation in the injured spinal cords of rats subjected to prolonged hind paw ischemia, a model of chronic post-ischemic pain, and mice with thoracic spinal contusion injury, has been previously reported to play a fundamental role in nociception [52, 53].

In the current study, CLZ abrogated the NLRP3 inflammasome assembly and activation to reduce ASC, pro-caspase-1, and IL1β, effects that are in line with previous data. In a model of human vascular endothelial cells, CLZ decreased ASC and caspase-1 [54], while in renal I/R, it suppressed caspase-1 [27], and in Parkinson-diseased rats, it reduced IL-1β [28]. CLZ by reducing Glu formation in the cerebral cortex with the downregulated NF-κB is the first reason for the NLRP3 inflammasome deactivation shown in the present work. In agreement with an earlier study in hypercholesterolemic rats, CLZ suppressed inhibitory kappa Bα degradation [55], whereas in renal I/R, it suppressed NF-κB [27]. These previous studies lend credence to the present anti-neuroinflammatory potential of CLZ and its capacity to inhibit the priming step of NLRP3 inflammasome activation by inhibiting Glu production.

Noteworthy, TRPA1 acts as an inflammation gatekeeper [56], hence propagating central nociception following the direct activation of NF-κB as shown previously in astrocytes stimulated by amyloid beta and TRPA1-transfected HEK293 cells [57] or by the indirect enhancement of glutamatergic transmission of nociceptive signals to increase hypersensitivity to painful stimuli as previously documented in the spinal cord by [58]. This ion channel is expressed in neurons across layers of the somatosensory cortex [59] and cerebral cortical astrocytes [60], and its activation arises from and contributes to the generation of ROS by activating NF-κB [57]. Moreover, Hatano et al. [61] reported that the TRPA1 promoter has NF-κB binding sites and its transcriptional induction by NF-κB in human synoviocytes; hence, the ability of CLZ to downregulate NF-κB can clarify the present suppression of TRPA1 as another factor that limits pain sensation. The present data support thus the role of brain TRPA1 deactivation in counteracting neuropathic modality as hyperalgesia in rats [62] and allodynia in mice [63]. Following the I/R injury, ROS is the third contributor to NLRP3 inflammasome activation thru detachment of the ubiquitous thioredoxin (TRX) and TRX-interacting protein complex [44]. Accordingly, it can be anticipated that CLZ by suppressing TRPA1 and ROS could intersect the NF-κB/ROS and TRPA1 vicious cycle to again deactivate the NLRP3 inflammasome assembly/activation dampening thus neuropathic pain.

In the present investigation, the antioxidant ability of CLZ could be a direct reason for the deactivation of NLRP3 inflammasome which was indeed verified by increased GSH to reduce the oxidative stress burden by virtue of its well-known free radical scavenging property [64]. The antioxidant potential of CLZ has been previously documented in different experimental settings [27, 28], an effect that is linked to the present CLZ-mediated SIRT-1 enhancement which has been reported to promote FOXO3a ubiquitination and degradation [65]. This increment in SIRT-1 thus affords an explanation for our observed decrease in FOXO3a that functions as a negative regulator of Nrf2 by promoting its degradation [66] to clarify the associated increase in the transcription factor justifying therefore the current GSH enhancement by CLZ. Nrf2 which acts as a master regulator of GSH is controlled by the active phosphorylation of Akt at Ser473, seen here and hitherto in models of oxidative hepatocellular toxicity [67] and focal cerebral ischemia [68]. Notably, Nrf2 nuclear translocation facilitates the transcription of antioxidant genes associated with GSH biosynthesis and cystine uptake [69]. Meanwhile, the same SIRT-1 signaling molecule can deactivate p65 NF-κB by its deacetylating capacity [70] to abrogate the formation of ROS, besides IL-1β and the NLRP3 inflammasome components documented herein after CLZ administration.

The analgesic efficacy of CLZ against thalamic pain, owing to the drug-mediated increment in cAMP via its PDE-3 inhibitory capacity, in a case report has been previously elucidated [29]. Indeed, this second messenger is also elevated by other PDE inhibitors that have been documented to suppress neuropathy. In this context, a PDE2A inhibitor reduced mechanical allodynia in non-compressive lumbar disc herniation rats via subsiding radicular inflammation [71], while a PDE4-specific inhibitor, lessened mechanical allodynia in a rat spinal nerve ligation model [72], effects attributable to the prevention of cAMP degradation. However, the present findings highlight the role of the DA/cAMP/CREB/BDNF/Akt in dampening the NLRP3 inflammasome action. This is contrary to the reduction in DA after the transient ischemic insult shown herein and in an earlier study [73] where the co-authors attributed such a decrement to the deactivation of tyrosine hydroxylase. On the other hand, CLZ post-treatment enhanced the neurotransmitter content to inhibit the NLRP3 inflammasome assembly/activation; hence, hyperalgesia/allodynia in this work. In a PD model, the drug elevated striatal DA by activating tyrosine hydroxylase [28] to lend credence to the present ability of CLZ to enhance the cortical monoamine content that acts as the fourth reason for NLRP3 inflammasome inhibition. Vice versa, Qiao et al. [74] reported that the inhibition of hepatic NLRP3 protected dopaminergic neurons in the brain providing thus a mutual relationship between DA and the NLRP3 inflammasome. Notably, by binding DA to D1 receptor [45]; besides, by CLZ well-documented pharmacological activity [26], the antiplatelet could increase the production of cAMP that directly stimulates the Akt signaling to enhance axonal outgrowth and nerve functional recovery which in turn diminishes neuropathic pain sensation [72]. By another mechanism, cAMP endorses the ubiquitination and degradation of NLRP3, a fundamental component of the inflammasome platform [75] to consolidate the present data in the treated rats.

A further consequence of enhanced cAMP/Akt signaling by CLZ, in the present work is CREB phosphorylation at Ser133; this transcription factor is a key player in multiple intracellular processes [76]. Indeed, the activation of the CREB pathway is involved in protection against several post-ischemic disorders including allodynia and hyperalgesia [77]. The current activation of CREB by CLZ treatment may account for the elevation of SIRT-1, BDNF, and TrkB as shown herein and hitherto [78, 79]. Apart from SIRT-1-induced protection, BDNF is a potent survival-promoting factor for sensory neurons responsive to temperature and tactile pain [80]. Moreover, it holds the capacity to alter nociception pain pathways reaching the brain [80], whereas ACC-specific deletion of BDNF exaggerated hyperalgesia in adult mice [81]*.* The current BDNF increment has been reported to aid in the activation of the Akt/CREB trajectory via its interaction with its receptor TrkB [82] providing a fifth reason for the NLRP3 inflammasome deactivation. In this context, the consequent phosphorylation/ activation of Akt at Ser473 results in the inhibition of NF-κB with the shutdown of the NLRP3 inflammasome as previously reported in myocardial I/R-exposed rats [83] to give further clarification to CLZ anti-nociceptive potential shown herein.

In this study, CLZ by rescuing GSH, DA, and BDNF and reducing Glu and TRPA1 can inhibit both NLRP3 inflammasome–mediated ASC and IL-1β production and release from pyroptotic neuronal cells or the released IL-1β by astrocytes. The augmented activation of the NLRP3 inflammasome incites NF-κB activity again by IL-1β receptor interaction [84]. Notably, Xu et al. [85] reported that following global cerebral I/R injury lysosomal rupture ensues which is critical for neuronal programmed necrosis that could be a reason for the release of ASC specks upon the rupture of lysosomes [86] with the mutual pyroptotic process to trigger NLRP3 inflammasome activation [87]. In astrocytes, NF-κB is responsible for both astrogliosis as indicated herein and earlier [88] by the elevation in GFAP and the secretion of IL-1β produced by NLRP3 inflammasome activation which then induces the secretion of further inflammatory cytokines from astrocytes [89] that modulates the excitatory NMDAR as formally reported in spinal neurons [90]. This suggests crosstalk between the activated astrocytes and NLRP3 inflammasome priming/assembly/activation in neurons. Astrocytes also harbor the TRAP1 [91] and IL-1β receptor [92], which upon activation continuously trigger the inflammasome activation in feed-forward cycles. In astrocytes, CLZ could have also mediated Nrf2 activation with the GSH synthesis/release to augment the antioxidant pool in nearby neurons [69]. Accordingly, the present capacity of CLZ in reducing astrogliosis and both inflammasome products (IL-1β and ASC) in association with its antioxidant character provides further mechanisms for the NLRP3 inflammasome disassembly/deactivation to curtail the previous cascades conferring thus analgesia and neuroprotection.

Hence, the current work introduces inhibition of the NLRP3 inflammasome priming/assembly/activation as a new indication for CLZ to combat post-ischemic pain through multiple targets. Primarily, CLZ anti-inflammatory and antioxidant potentials mediated by modulating cortical Glu, DA, and TRPA1/NLRP3 inflammasome signaling verifies its capacity to treat post-ischemic neuropathic pain. These findings add a beneficial role for the drug on top of its known antiplatelet effect against painful cerebral I/R consequences. However, further studies are required to extend these animal model findings and the pathogenesis to humans with central post-ischemic pain.

## Supplementary Information

Below is the link to the electronic supplementary material.

Supplementary file1 (DOCX 458 KB)

## Data Availability

The datasets generated and/or analyzed during the current study are available from the corresponding author upon reasonable request.

## Abbreviations

ABC: Avidin-biotin complex
ACC: Anterior cingulate cortex
Akt: Protein kinase B
ASC: Apoptosis-associated speck-like protein
BDNF: Brain-derived neurotrophic factor
cAMP: Cyclic adenosine monophosphate
Casp-1: Cysteine aspartate-specific protease-1
CCAs: Common carotid arteries
CLZ: Cilostazol
CREB: CAMP responsive element binding
DA: Dopamine
DAB: Diaminobenzidine tetrahydrochloride
FOXO3a: Forkhead box protein O3a
GFAP: Glial fibrillary acidic protein
Glu: Glutamate
GSH: Glutathione
H&E: Hematoxylin and eosin
I/R: Ischemia/ reperfusion
IL-1β: Interleukin-1 beta
NLRP3: Nucleotide-binding domain-like receptor protein 3
NMDAR: *N*-Methyl-d-aspartate receptor
Nrf2: Nuclear factor E2-related factor or nuclear factor (erythroid-derived 2)-like 2
p65 NF-κB: P65 Nuclear factor kappa B
PBS: Phosphate buffer saline
PDE-3: Phosphodiesterase-3
PWL: Paw withdrawal latency
PWT: Paw withdrawal threshold
RIPA: Radioimmunoprecipitation assay
ROS: Reactive oxygen species
SIRT-1: Sirtuin-1
SO: Sham-operated
TBST: Tris-buffered saline containing Tween
TrkB: Tropomyosin receptor kinase B
TRPA1: Transient receptor potential ankyrin 1
TRX: Thioredoxin
