# Supplementary material for: Cilostazol Alleviates NLRP3 Inflammasome–Induced Allodynia/Hyperalgesia in Murine Cerebral Cortex Following Transient Ischemia: Focus on TRPA1/Glutamate and Akt/Dopamine/BDNF/Nrf2 Trajectories
Source: Mol Neurobiol. 2022 Sep 20;59(12):7194–211. doi: 10.1007/s12035-022-03024-w (PMC9616778; doi:10.1007/s12035-022-03024-w)
Supplement: Supplementary file 1 — Supplementary file1 (DOCX 458 KB) [file 12035_2022_3024_MOESM1_ESM.docx]

**Cilostazol alleviates NLRP3 inflammasome-induced allodynia/hyperalgesia**

**via modulating TRPA1/glutamate and Akt/dopamine/BDNF/Nrf2 trajectories**

**in murine cerebral cortex following transient ischemia: Insights into post- stroke neuropathic pain**

**Journal name: Molecular Neurobiology**

**Omnia S. Zaki^1^, Noha N. Nassar^2^, Dalaal M. Abdallah*^2^, Marwa M. Safar^2,3^, Reham A. Mohammed^2^**

^1^Department of Pharmacology and Toxicology, Faculty of Pharmacy, Modern University for

Technology and Information, Cairo, Egypt

^2^Department of Pharmacology and Toxicology, Faculty of Pharmacy, Cairo University, Cairo,

Egypt

^3^Department of Pharmacology and Biochemistry, Faculty of Pharmacy, The British University in

Egypt, Cairo, Egypt

***Corresponding author**

Dalaal M. Abdallah, Prof.

Address: Department of Pharmacology and Toxicology, Faculty of Pharmacy, Cairo University,

Kasr El-Aini Street, Cairo 11562, Egypt.

E-mail: dalaal. [abdallah@pharma.cu.edu](mailto:abdallah@pharma.cu.edu).

**Cleaved Casp-1**

**a**

**SO**

**I/R+CLZ**

**I/R**

**SO+CLZ**

**
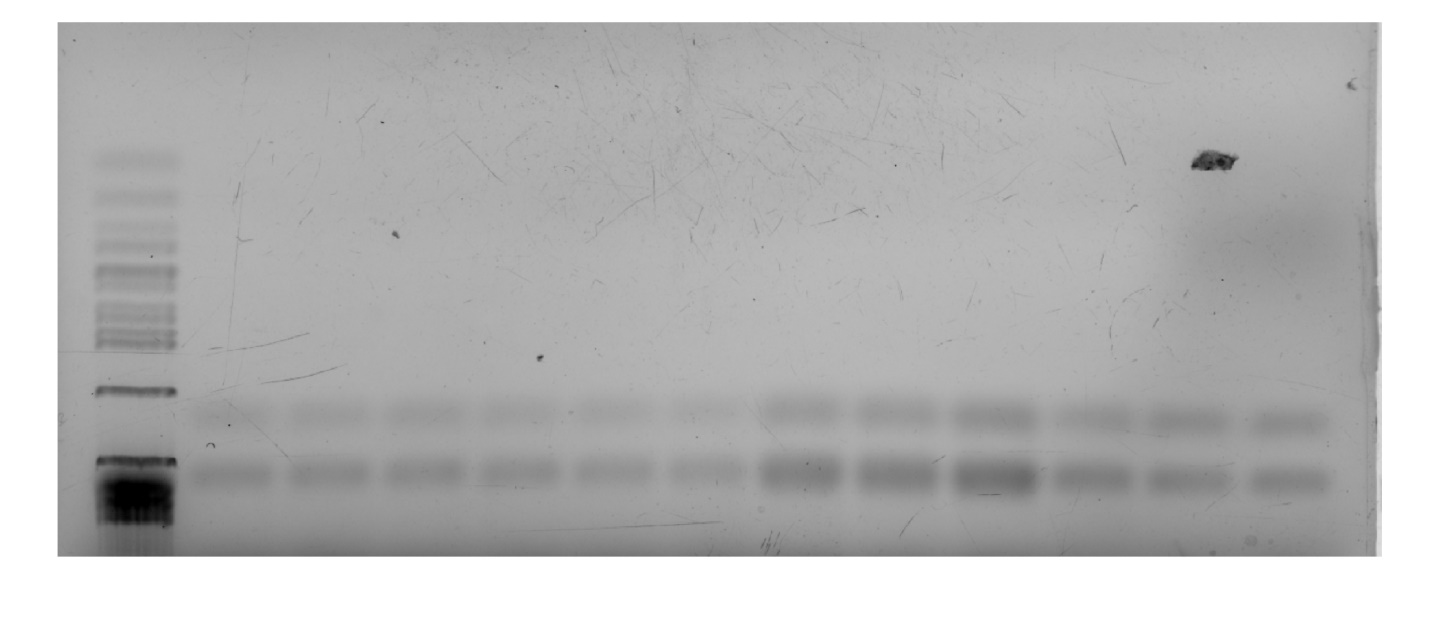
**

**Total Casp-1**

**I/R+CLZ**

**SO+CLZ**

**I/R**

**SO**


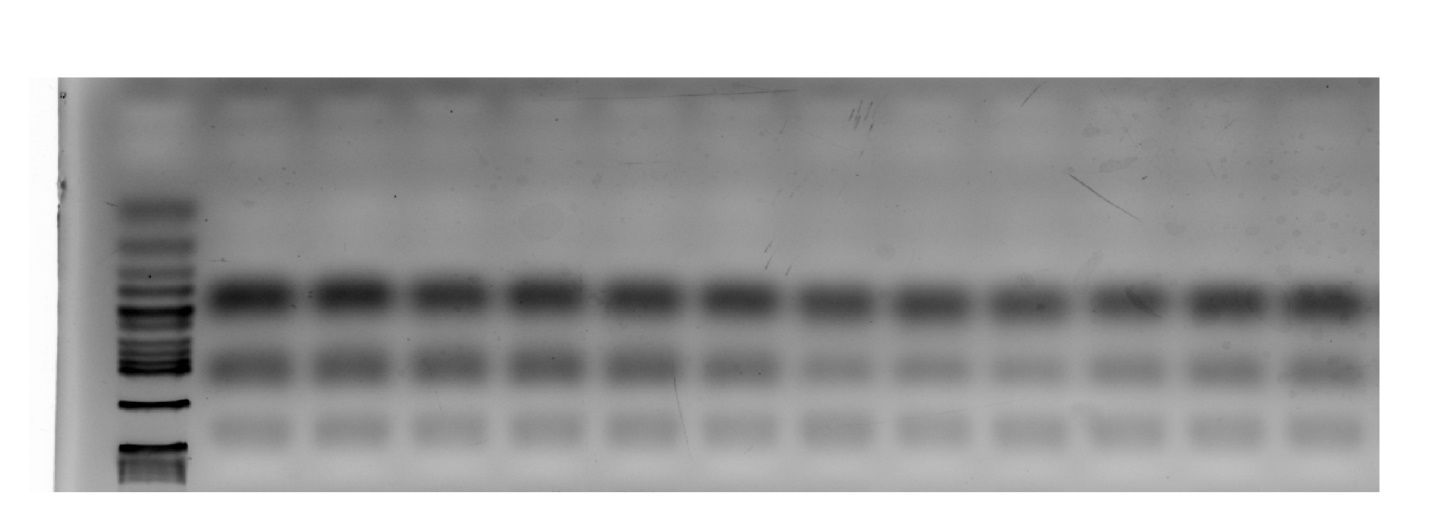


**Foxo3a**

**b**

**SO+CLZ**

**I/R**

**I/R+CLZ**

**SO**


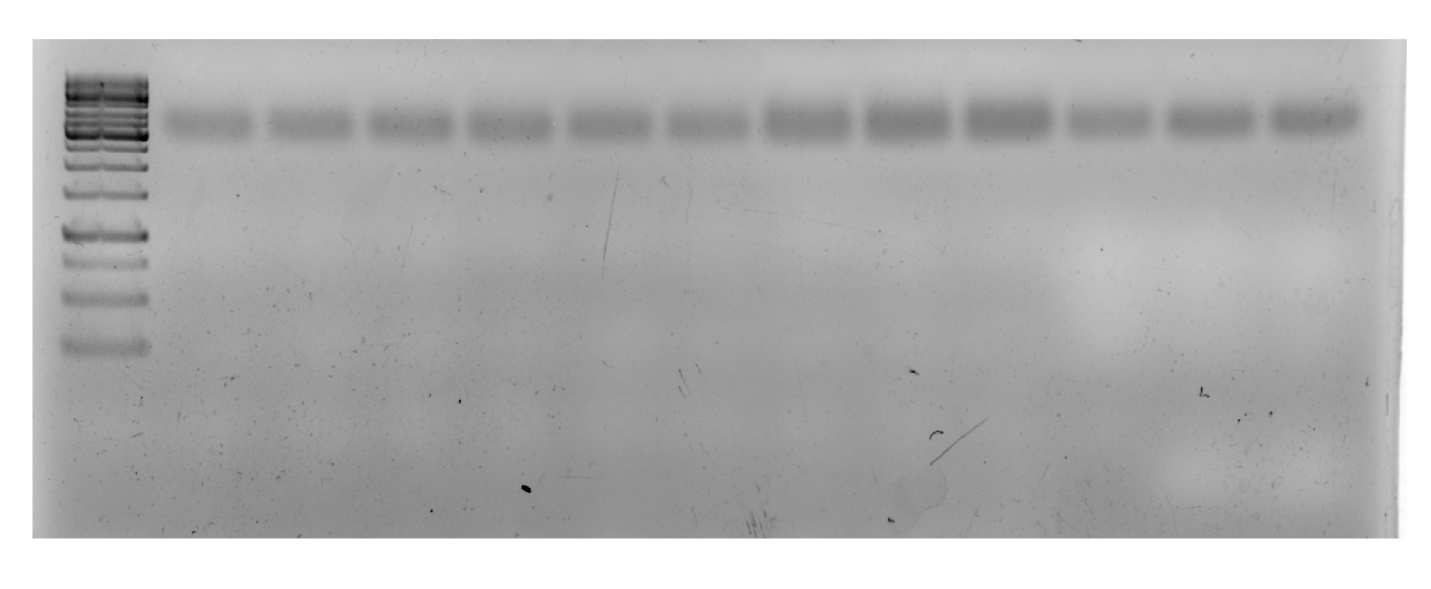


**β-actin**

**I/R+CLZ**

**I/R**

**SO**

**SO+CLZ**


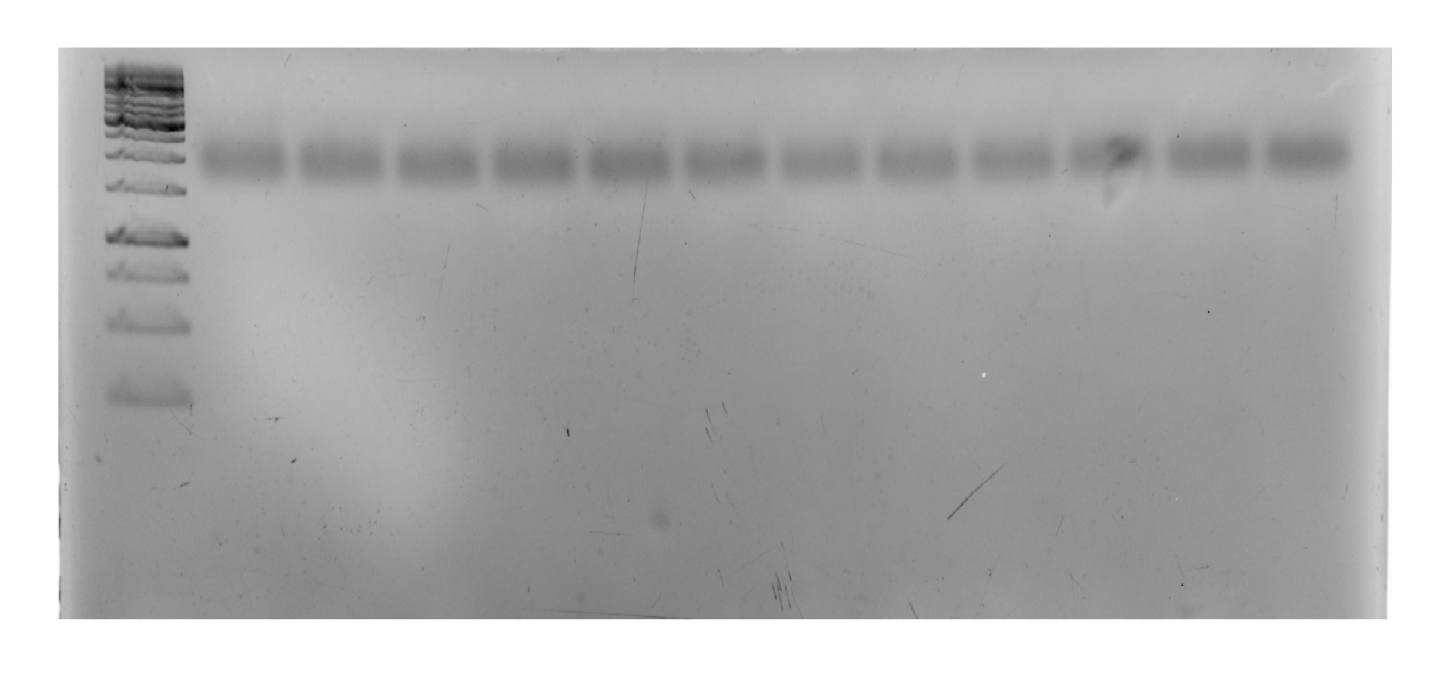


**Supplementary Fig. S1: Original Western blot images for Figures.** (a) Uncropped Western blot for Fig. 4bii. (b) Uncropped Western blot for Fig. 6b. Following β-actin staining of a and b membranes. CLZ: cilostazol; Casp-1: caspase-1; FOXO3a: forkhead box O3a; I/R: ischemia/reperfusion; SO: sham-operation
